# ROMO1 is a constituent of the human presequence translocase required for YME1L protease import

**DOI:** 10.1083/jcb.201806093

**Published:** 2019-02-04

**Authors:** Frank Richter, Sven Dennerlein, Miroslav Nikolov, Daniel C. Jans, Nataliia Naumenko, Abhishek Aich, Thomas MacVicar, Andreas Linden, Stefan Jakobs, Henning Urlaub, Thomas Langer, Peter Rehling

**Affiliations:** 1 Department of Cellular Biochemistry, University Medical Center Göttingen, Göttingen, Germany; 2 Bioanalytical Mass Spectrometry Group, Max Planck Institute for Biophysical Chemistry, Göttingen, Germany; 3 Department of NanoBiophotonics, Mitochondrial Structure and Dynamics Group, Max Planck Institute for Biophysical Chemistry, Göttingen, Germany; 4 Department of Neurology, University Medical Center, Göttingen, Germany; 5 Department of Mitochondrial Proteostasis, Max Planck Institute for Biology of Ageing, Cologne, Germany; 6 Bioanalytics Group, Department of Clinical Chemistry, University Medical Center Göttingen, Göttingen, Germany; 7 Max Planck Institute for Biophysical Chemistry, Göttingen, Germany

## Abstract

The mitochondrial presequence translocation machinery (TIM23 complex) is conserved between the yeast *Saccharomyces cerevisiae* and humans; however, functional characterization has been mainly performed in yeast. Here, we define the constituents of the human TIM23 complex using mass spectrometry and identified ROMO1 as a new translocase constituent with an exceptionally short half-life. Analyses of a ROMO1 knockout cell line revealed aberrant inner membrane structure and altered processing of the GTPase OPA1. We show that in the absence of ROMO1, mitochondria lose the inner membrane YME1L protease, which participates in OPA1 processing and ROMO1 turnover. While ROMO1 is dispensable for general protein import along the presequence pathway, we show that it participates in the dynamics of TIM21 during respiratory chain biogenesis and is specifically required for import of YME1L. This selective import defect can be linked to charge distribution in the unusually long targeting sequence of YME1L. Our analyses establish an unexpected link between mitochondrial protein import and inner membrane protein quality control.

## Abstract (precis)

Mitochondria are the powerhouses of eukaryotic cells and rely on protein import from the cytosol. Richter et al. found ROMO1 as a new constituent of the human mitochondrial import machinery linking protein import to quality control and mitochondrial morphology.

## Introduction

Mitochondria are of central importance for the metabolism of eukaryotic cells such as the generation of ATP by oxidative phosphorylation. Moreover, mitochondria are recognized for their roles in signaling processes and apoptosis (McBride et al., 2006; Chacinska et al., 2009; Nunnari and Suomalainen, 2012; Harbauer et al., 2014; Sokol et al., 2014). Mitochondria possess a characteristic double-membrane morphology. The inner membrane is folded into cristae, which house the oxidative phosphorylation system (Wiedemann and Pfanner, 2017). At the cellular level, mitochondria form a highly dynamic network that undergoes constant fission and fusion events (Westermann, 2010; MacVicar and Langer, 2016). A crucial regulator of mitochondrial dynamics and structure is the dynamin-related GTPase optic atrophy type 1 (OPA1) in the inner membrane (Delettre et al., 2000). OPA1 is processed by the ATP-dependent YME1L protease and the zinc metalloprotease OMA1 (Ishihara et al., 2006; Song et al., 2007; Anand et al., 2014). These proteases balance long (L)-OPA1 and short (S)-OPA1 forms. Maintaining a proper ratio between L- and S-OPA1 is required for maintenance of mitochondrial morphology (Anand et al., 2014). Moreover, YME1L facilitates the turnover of a number of inner membrane and intermembrane space proteins, such as the translocase components TIM23 and TIM17A (Rainbolt et al., 2013; Wai et al., 2016) and the lipid transfer proteins PRELID1 and STARD7 (Potting et al., 2013; Saita et al., 2018) and is important for mitochondrial protein quality.

**Figure 1.**
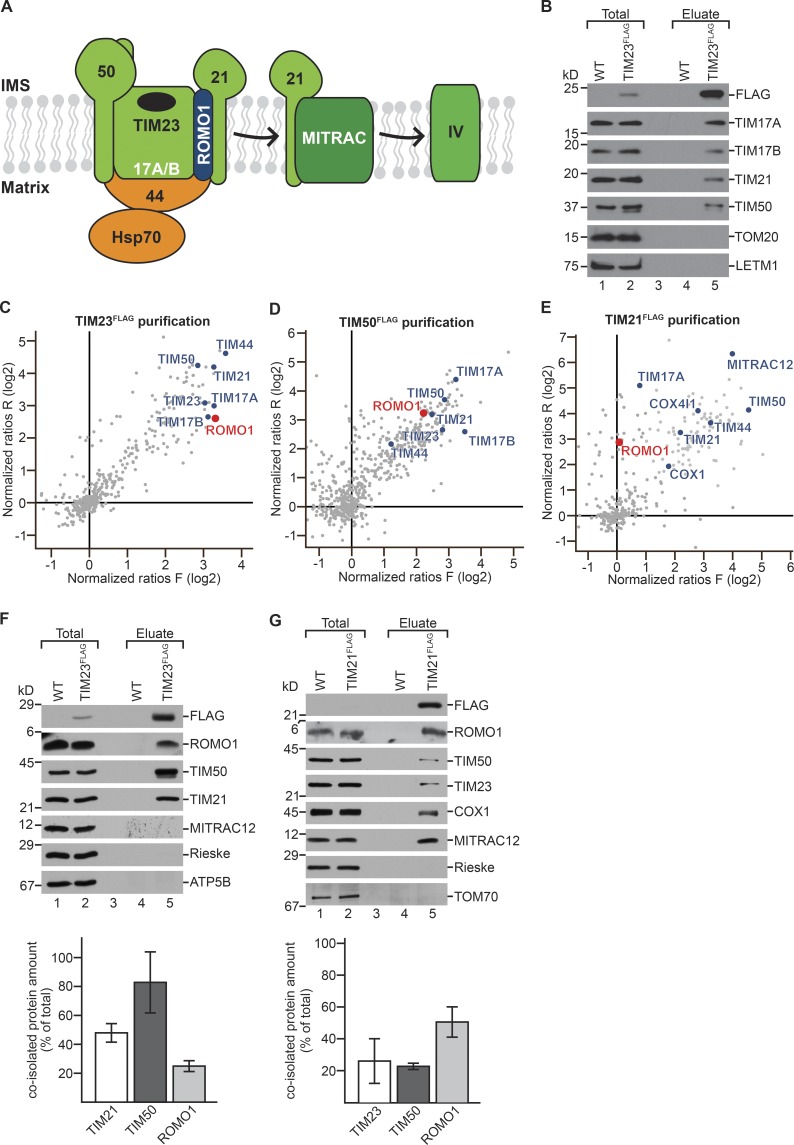
**ROMO1 is a component of the TIM23 complex. (A)** Scheme of the human presequence translocase/TIM23 complex consisting of TIM23, TIM17A/B, TIM21, TIM50, and ROMO1 as well as TIM44 and HSP70. TIM21 not only associates with the TIM23 complex but also with the complex IV assembly factor MITRAC. **(B)** HEK293T WT and TIM23^FLAG^ mitochondria were solubilized in digitonin and incubated with anti-FLAG beads, eluted by FLAG-peptide, and analyzed by SDS-PAGE and immunoblotting using the indicated antibodies. Eluate: 100%, total: 1.5%. **(C–E)** Scatter plots representing the normalized H/L protein ratios (log2) of the forward (F) and reversed (R) SILAC experiments after MS analyses of TIM23^FLAG^ (C), TIM50^FLAG^ (D), and TIM21^FLAG^ (E) immunoprecipitations. **(F)** HEK293T WT and TIM23^FLAG^ mitochondria were solubilized in digitonin and incubated with anti-FLAG beads, eluted by FLAG-peptide, and analyzed by SDS-PAGE and immunoblotting using the indicated antibodies. Eluate: 100%, total: 1.6%. Results are presented as quantification of eluate/total × factor of elution/total (62.5; means ± SEM, *n* = 3). **(G)** WT and TIM21^FLAG^ mitochondria were analyzed as described in F. Eluate: 100%, total: 1.6%. Results are presented as quantification of eluate/total × factor of elution/total (62.5; means ± SEM, *n* = 3).

Most mitochondrial proteins are imported from the cytosol in a signal-dependent manner and enter mitochondria through the TOM complex (Neupert and Herrmann, 2007; Chacinska et al., 2009; Mokranjac and Neupert, 2010; Dudek et al., 2013; Sokol et al., 2014; Wiedemann and Pfanner, 2017; Kang et al., 2018). N-terminal presequences are signals used by 60% of mitochondrial proteins, and they direct these to mitochondria and across the inner mitochondrial membrane aided by the presequence translocase (TIM23 complex; Fig. 1 A; Vögtle et al., 2009; Schulz et al., 2015; Wiedemann and Pfanner, 2017). Presequences are usually 15–50 amino acids long and processed upon import. Initial translocation of the presequence across the inner membrane is driven by the mitochondrial membrane potential (Δψ; Schleyer et al., 1982; Roise and Schatz, 1988; Martin et al., 1991; Chacinska et al., 2009; Schulz et al., 2015; Wiedemann and Pfanner, 2017). The TIM23 complex facilitates the transport of proteins into the matrix and the insertion of membrane proteins into the lipid phase (protein sorting). While the Δψ suffices to drive transport of precursors that are sorted into the inner membrane, matrix protein transport requires the activity of the ATP-driven presequence translocase-associated motor (PAM complex; Neupert and Brunner, 2002; Frazier et al., 2004; van der Laan et al., 2007; Schulz and Rehling, 2014; Wiedemann and Pfanner, 2017).

The yeast presequence translocase consists of the pore-forming Tim23 and Tim17 and the presequence receptor Tim50 (Dekker et al., 1997; Truscott et al., 2001; Geissler et al., 2002; Yamamoto et al., 2002; Chacinska et al., 2003, 2005; Mokranjac et al., 2003; Meinecke et al., 2006; Schulz et al., 2011). Tim21 couples the translocase to the respiratory chain and interacts with the TOM complex (Chacinska et al., 2005; Mokranjac et al., 2005; van der Laan et al., 2006). The integral membrane protein Mgr2 has been suggested to perform multiple functions at the TIM23 complex. Mgr2 apparently recruits Tim21 to the TIM23 complex. In addition, it provides a yet undefined quality control function during lateral protein transport as indicated by the observation that *mgr2* mutant cells display a faster membrane insertion of precursors into the inner membrane than the WT. Mgr2 appears to be involved in the dynamic recruitment of the import motor (Gebert et al., 2012; Ieva et al., 2014; Schulz and Rehling, 2014). Recent work shows that at the Tim23 channel, the presence of the import motor or Mgr2 are mutually exclusive since the import motor component Pam18 blocks the lateral gate of the channel and thereby appears to block access of Mgr2 to the translocase (Schendzielorz et al., 2018). In yeast, the key component of the import motor is the mitochondrial Hsp70 (mtHsp70), which is regulated by chaperones Pam16/18 and Mge1 and recruited to the translocase by Tim44 (Chacinska et al., 2009; Schulz et al., 2015; Wiedemann and Pfanner, 2017). While general principles of protein translocation are conserved in humans (Bauer et al., 1999; Sokol et al., 2014; Kang et al., 2018), there appear to be substantial functional differences. For example, Tim17 is present in two paralogs, TIM17A and TIM17B (Fig. 1 A). Interestingly, TIM17A is turned over under stress in a YME1L-dependent manner (Rainbolt et al., 2013). Human TIM21 associates with the TIM23 complex but is dispensable for protein import (Mick et al., 2012). In addition, it is a constituent of the MITRAC complex, which represents an assembly intermediate of cytochrome *c* oxidase (Fig. 1 A). TIM21 ushers newly imported respiratory chain subunits (e.g., COX4I-1) to the assembly intermediate that harbors the mitochondrial-encoded COX1 subunit, thereby linking protein import to respiratory chain assembly (Mick et al., 2012; Fig. 1 A).

Here, we defined the composition of the human TIM23 complex and identified ROMO1 as a novel subunit. ROMO1 displays sequence similarity to yeast Mgr2 (Fig. S1 A). Previous studies correlated high levels of ROMO1 with increased reactive oxygen species (ROS) production in cancer cells and tissue (Chung et al., 2006, 2008; Na et al., 2008; Shin et al., 2013; Wu et al., 2015). Furthermore, ROMO1 has been implicated in regulation of OPA1 processing and mitochondrial dynamics (Norton et al., 2014) and shown to exert nonselective cation channel activity in vitro (Lee et al., 2018). Here, we elucidate the molecular function of ROMO1 in mitochondrial protein import. ROMO1 is a protein with an exceptionally short half-life that couples TIM21 to the presequence translocase. We find that ROMO1 is dispensable for general import processes but specifically required for the import of newly synthesized YME1L. The ROMO1 dependence for import is due to the low number of positively charged amino acids in the presequence of YME1L. While ROMO1 is involved in the import of YME1L, YME1L degrades ROMO1, thus limiting its own import. Our data therefore establish an unexpected link between protein import, protein quality control, and inner membrane morphology.

## Results

### ROMO1 is a component of the TIM23 complex

To define the composition of the human TIM23 complex, we set out to purify the human presequence translocase. To this end, we generated a stable HEK293T cell line, using a genomically integrated TIM23^FLAG^-encoding cassette enabling protein expression at physiological levels. Natively immunoisolated TIM23^FLAG^-containing complexes were first analyzed by Western blotting. As expected, known TIM23 complex constituents such as TIM17A, TIM17B, TIM21, and TIM50 were identified in the eluate (Fig. 1 B). Based on this finding, we also generated stable HEK293T cell lines with genomically integrated TIM50^FLAG^- and TIM21^FLAG^-encoding cassettes. Subsequently, we purified the TIM23 complex via TIM23^FLAG^, TIM50^FLAG^, and TIM21^FLAG^ using differential stable isotope labeling with amino acids in cell culture (SILAC; Ong et al., 2002) and performed mass spectrometry (MS) analyses. In these, we recovered expected subunits of the TIM23 complex such as TIM17A, TIM17B, and TIM44 with TIM23, TIM21, and TIM50 (highlighted in Fig. 1, C–E). Moreover, in case of TIM21^FLAG^, subunits of the MITRAC assembly intermediate were identified (highlighted in Fig. 1 E). This was expected, since TIM21 shuttles between the translocase and MITRAC assembly intermediates of COX1 (Mick et al., 2012). Moreover, in all analyses, we identified a protein named ROMO1 (ROS modulator 1; Chung et al., 2006).

We confirmed the MS data by immunoisolations and Western blot analyses. TIM23^FLAG^ efficiently copurified ROMO1 together with TIM21 and TIM50, while other inner membrane proteins associated with the OXPHOS machinery, i.e., Rieske and ATP5B, were not recovered (Fig. 1 F). Quantifications showed that 25% of ROMO1 copurified with TIM23^FLAG^. Moreover, ROMO1 was efficiently coisolated with TIM21^FLAG^ along with TIM50, TIM23, COX1, and MITRAC12 (Fig. 1 G). Quantifications showed that ∼50% of mitochondrial ROMO1 copurified with TIM21^FLAG^, while TIM23 and TIM50 coisolated with slightly lower efficiencies. This is in agreement with previous findings that TIM21 not only functions at the translocase but is also a MITRAC complex constituent (Mick et al., 2012). We conclude that ROMO1 is a new constituent of the human presequence translocase (Fig. 1 A). Moreover, our analyses show that under these experimental conditions not the entire ROMO1 pool is translocase associated.

### Phenotypic analyses of loss of ROMO1 in knockout and siRNA-treated cells

**Figure 2.**
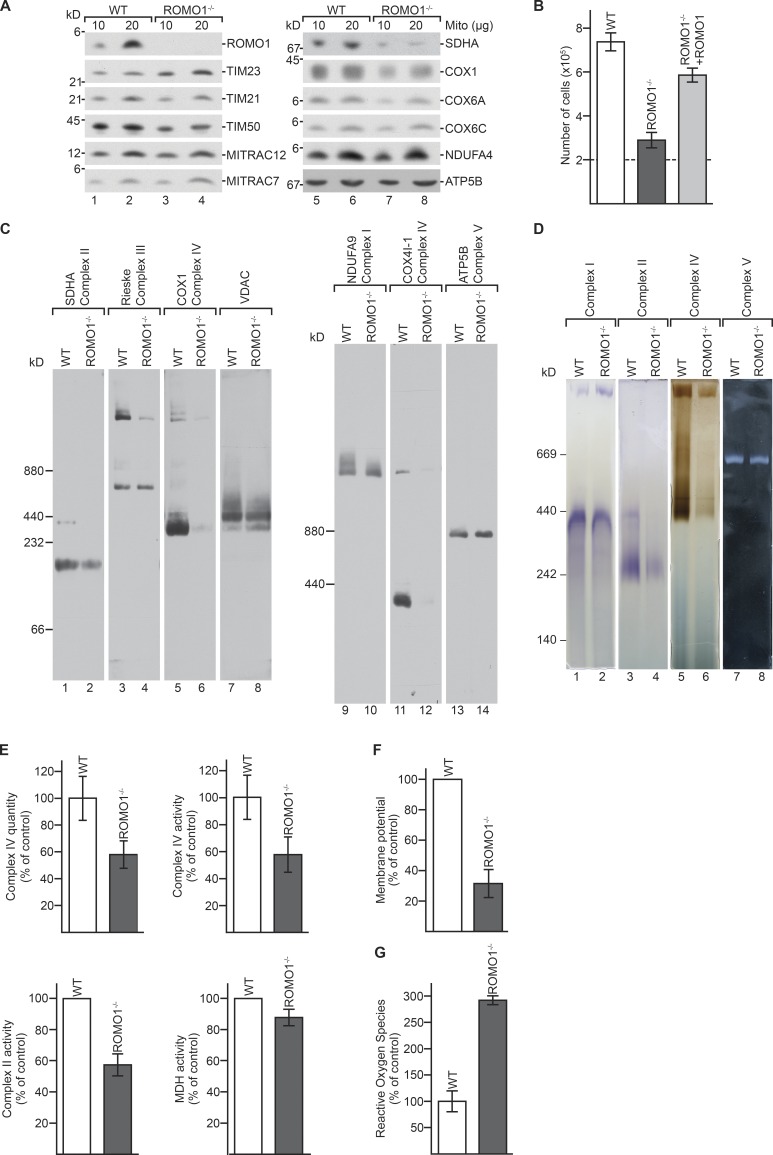
**ROMO1^−/−^ cells display increased ROS production**. **(A)** Mitochondria from HEK293T WT and ROMO1^−/−^ cells were lysed and analyzed by SDS-PAGE and Western blot. **(B)** Cell proliferation of WT, ROMO1^−/−^, and ROMO1^−/−^ cells expressing ROMO1 under control of a tetracycline-inducible promoter. Dashed line represents starting cell number (means ± SEM, *n* = 3). **(C)** Mitochondria from WT and ROMO1^−/−^ cells were solubilized in digitonin and analyzed by BN-PAGE and immunoblotting using indicated antibodies. **(D)** Mitochondria were lysed as in C, analyzed by BN-PAGE and in-gel activity assays for complexes I, II, IV, and V. **(E)** Measurements of enzyme quantity and activity of complex IV of solubilized and immobilized WT and ROMO1^−/−^ cells, enzyme activity of complex II of WT and ROMO1^−/−^ mitochondria, and malate dehydrogenase (MDH) activity of solubilized WT and ROMO1^−/−^ mitochondria (means ± SEM, *n* = 3). **(F)** Measurement of membrane potential in WT and ROMO1^−/−^ cells using JC-1 dye (means ± SEM, *n* = 3). **(G)** ROS production was measured in whole cells using MitoSOX Red Mitochondrial Superoxide Indicator (means ± SEM, *n* = 3).

To investigate the function of ROMO1, we generated a ROMO1 knockout cell line (ROMO1^−/−^) using CRISPR/Cas9 technology. For this, we targeted the first exon and confirmed disruption of the genomic ORF by sequencing (Fig. S1 B). Furthermore, lack of ROMO1 was confirmed at the protein level by SDS-PAGE and immunoblotting comparing WT and ROMO1^−/−^ mitochondria. While in ROMO1^−/−^ mitochondria the amounts of several proteins, such as MITRAC12, MITRAC7, ATP5B, and TIM21, were unaffected, we observed increased levels of TIM23. Interestingly, TIM50, SDHA, COX1, COX6A, and COX6C levels were decreased (Fig. 2 A). To investigate the effect of loss of ROMO1 at the cellular level, we analyzed the growth rate of ROMO1^−/−^ cells. A significant growth reduction was apparent in the ROMO1 deletion strain. This growth phenotype could be rescued by expressing ROMO1 in ROMO1^−/−^ cells (ROMO1^−/−^ + ROMO1; Fig. 2 B).

Considering the observed reduced steady state levels of respiratory chain subunits (Fig. 2 A), we analyzed the oxidative phosphorylation system in WT and ROMO1^−/−^ cells by Blue Native (BN)–PAGE and immunoblotting. Loss of ROMO1 led to a drastic decrease of complex IV. Furthermore, a decrease of the succinate dehydrogenase complex was observed, while complex I, monomeric complex III, complex V, and the outer membrane VDAC complex were only marginally affected (Fig. 2 C). These analyses were corroborated by in-gel activity staining of complex I, II, IV, and V, confirming the Western blot data. The activity of complex IV and II was clearly reduced (Fig. 2 D). Moreover, activity assays, using malate dehydrogenase activity as a control, revealed 40% reduction of both complex IV and II in ROMO1^−/−^ mitochondria (Fig. 2 E). Furthermore, the membrane potential was measured showing a 70% reduction (Fig. 2 F). This could explain the observed lower levels of imported proteins such as TIM50, COX6A, and COX6C. Conflicting data have been reported as to how ROMO1 expression correlates with mitochondrial ROS generation. While in some reports, a decrease of ROS correlated with decreasing ROMO1 expression (i.e., Chung et al., 2008; Lee et al., 2011; Shin et al., 2013; Shyamsunder et al., 2015), Norton et al. (2014) showed a twofold increase in ROS production upon siRNA-mediated reduction of ROMO1. This discrepancy prompted us to investigate ROS generation in ROMO1^−/−^ cells. In ROMO1^−/−^ cells, we observed a threefold increase of ROS in comparison to the control (Fig. 2 G).

**Figure 3.**
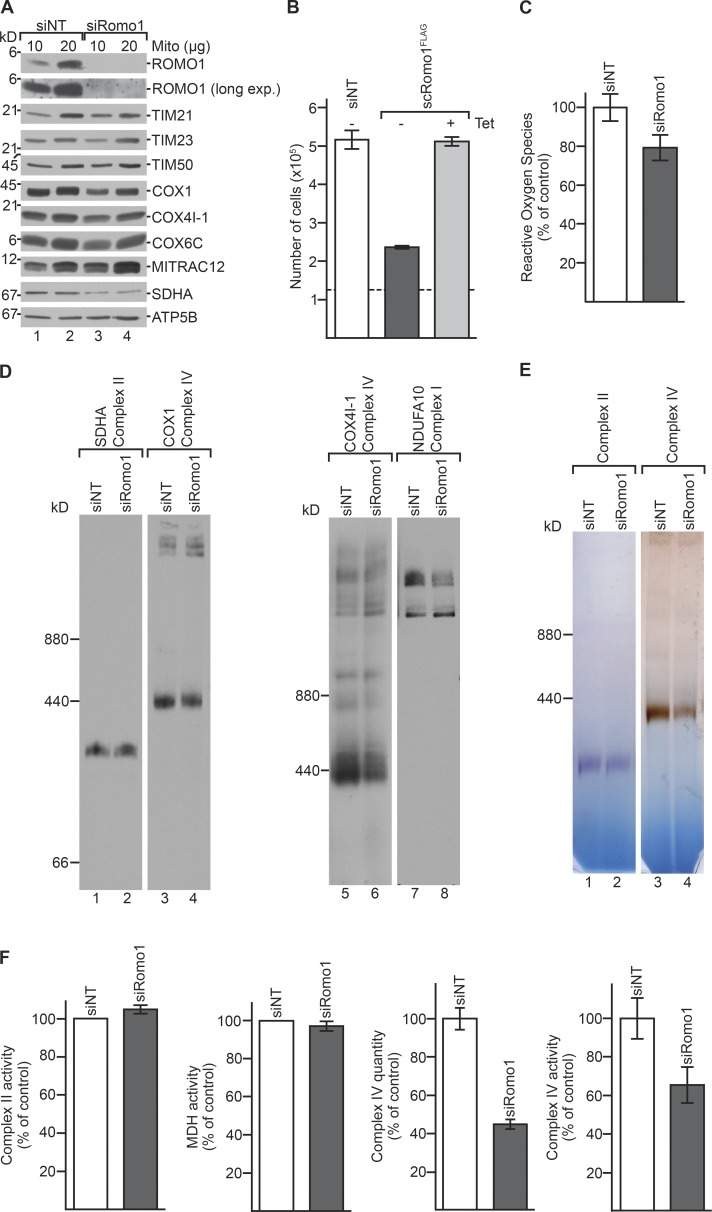
**Transient depletion of ROMO1 affects OXPHOS complexes but does not increase ROS. (A)** WT cells were treated with siRNA oligonucleotides against *Romo1* or nontargeting control siRNA (NT) for 72 h. Isolated mitochondria were lysed and analyzed by SDS-PAGE and Western blot. **(B)** Proliferation of cells inducibly expressing siRNA-resistant ROMO1^FLAG^ (scROMO1^FLAG^) treated with siNT or siRomo1 in the presence or absence of tetracycline (Tet). Dashed line represents starting cell number (means ± SEM, *n* = 3). **(C)** ROS production was measured in whole cells using MitoSOX Red Mitochondrial Superoxide Indicator (means ± SEM, *n* = 3). **(D)** siNT or siRomo1 mitochondria were solubilized in digitonin and analyzed by BN-PAGE and immunoblotting using indicated antibodies. **(E)** Mitochondria were lysed as in D and analyzed by BN-PAGE and in-gel activity assays for complexes II and IV. **(F)** Measurements of enzyme activity of complex II of siNT and siRomo1 mitochondria, malate dehydrogenase activity of solubilized siNTand siROMO1 mitochondria, and enzyme quantity and activity of complex IV of solubilized and immobilized siNT and siRomo1 cells (means ± SEM, *n* = 3).

The contradicting reports on mitochondrial ROS production upon ROMO1 depletion and our observations in ROMO1^−/−^ cells (Fig. 2 G) suggested that for assessment of ROMO1 function, we had to consider and avoid potential secondary effects caused by long-term loss of ROMO1. Silencing of ROMO1 was almost complete, as the protein could not be detected (Fig. 3 A). In contrast to ROMO1^−/−^ cells, upon siRNA-mediated ROMO1 depletion, we did not observe alterations in the amount of mitochondrial translocase components (e.g., TIM21, TIM23, and TIM50), while COX1, COX4I-1, and SDHA were decreased (Fig. 3 A). However, we observed similar growth reduction of siRNA depleted cells compared with ROMO1^−/−^ cells, which could be restored after expression of an siRNA-resistant version of ROMO1 (scROMO1^FLAG^; Fig. 3 B). To this end, we analyzed mitochondrial ROS production in ROMO1-depleted cells using siRNA-mediated knockdown. A slight decrease in ROS production was apparent when ROMO1 levels were reduced by siRNA-mediated knockdown, supporting the idea that increased ROS production was rather an indirect effect of ROMO1 deficiency (Fig. 3 C). In addition to the difference observed for ROS production, after transient ROMO1 depletion, complex II levels were not affected in the knockdown situation (Fig. 3 D). In contrast, similar to the defect observed in ROMO1^−/−^ mitochondria, complex IV levels were reduced upon ROMO1 depletion (Fig. 3 D). This was confirmed by in-gel activity assays, in which complex II did not show a reduction in activity, while complex IV activity was reduced compared with siNT (Fig. 3 E). Enzyme activity measurements confirmed that complex II activity was not significantly reduced while we measured a significant reduction of complex IV amount and activity using an immunoabsorption-based assay to quantify complex IV amount and activity (Fig. 3 F). We concluded that the phenotypes observed upon transient ROMO1 depletion reflected a more specific phenotype than what was seen in ROMO1^−/−^ cells.

### ROMO1 affects the distribution of TIM21 between TIM23 and MITRAC complexes

**Figure 4.**
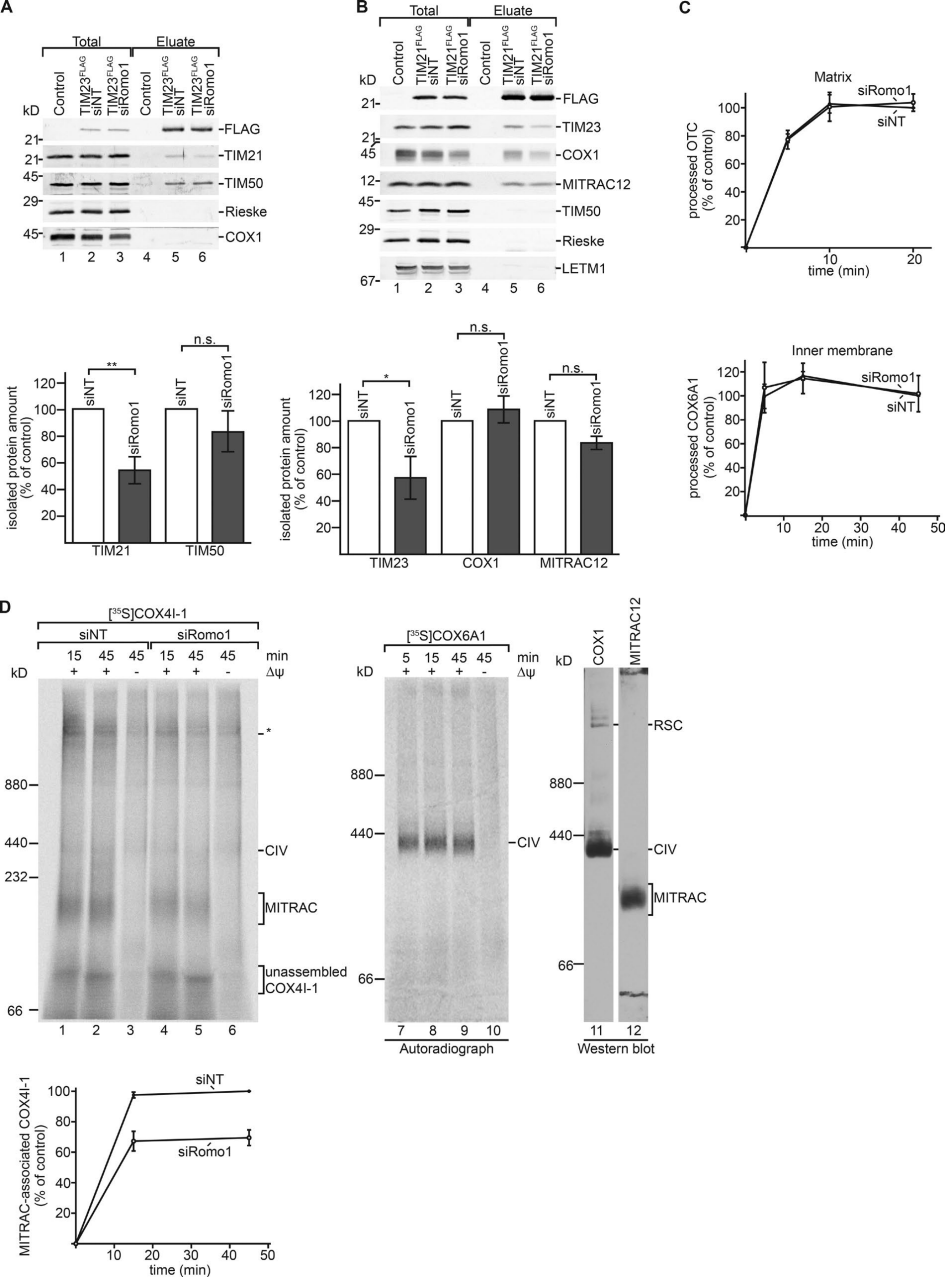
**ROMO1 affects the distribution of TIM21. (A and B)** HEK293T WT and TIM23^FLAG^ or TIM21^FLAG^ cells were treated with siRNA oligonucleotides against *Romo1* or nontargeting control siRNA (NT) for 72 h. Cells were solubilized in digitonin and incubated with anti-FLAG beads, eluted by FLAG-peptide, and analyzed by SDS-PAGE and immunoblotting using the indicated antibodies. Eluate: 100%, total: 5%. Results are presented as quantification of eluate/total normalized to efficiency of immunoprecipitation (FLAG signal in eluate). Values of siNT were set to 100% (means ± SEM, *n* = 3; **, P < 0.01; *, P < 0.05). **(C)** Indicated ^35^S-labeled precursors were imported into isolated energized mitochondria. Import of siNT sample at the longest time point was set to 100% (means ± SEM; *n* = 3) is shown. **(D)** [^35^S]COX4I-1 and [^35^S]COX6A1 precursors were imported into isolated energized mitochondria. Import was stopped at given time points by addition of antimycin A, valinomycin, and oligomycin (AVO). Samples were analyzed by BN-PAGE and autoradiography. WT mitochondria were solubilized in digitonin and analyzed by BN-PAGE and immunoblotting using indicated antibodies. MITRAC-associated COX4I-1 of siNT sample at the longest time point was set to 100% (means ± SEM; *n* = 4). CIV, complex IV; RSC, respiratory chain supercomplexes; *, undefined, Δψ independent complex.

Although the channel-forming TIM23 and TIM17 are core subunits of the translocase, TIM21 switches between TIM23- and MITRAC-complex association. To this end, we assessed whether ROMO1 influenced TIM21 distribution and analyzed the interactions between TIM23 and TIM21 upon siRNA-mediated ROMO1 depletion. Copurification of TIM21 with TIM23^FLAG^ was reduced in the absence of ROMO1 by ∼45%, while the amount of TIM50 remained unaffected (Fig. 4 A). Similarly, a reduced association between TIM21^FLAG^ and TIM23 was observed when TIM21^FLAG^ was used as bait. In contrast, the interactions to other TIM21 interactors, such as COX1 or MITRAC12 (Mick et al., 2012), were not significantly altered (Fig. 4 B). We concluded that ROMO1 participates in coupling TIM21 to the TIM23 complex.

The observed association of ROMO1 with the presequence translocase led us to analyze whether loss of ROMO1 affected import of presequence-containing matrix and inner membrane proteins. For this, we performed in vitro import of radiolabeled precursor proteins into control and ROMO1 knockdown mitochondria. Import of the matrix protein ornithine transcarbomyolase or the model matrix protein Su9-dihydrofolate reductase was not affected by loss of ROMO1 (Figs. 4 C and S2, A and B). Furthermore, import of the mitochondrial inner membrane proteins EMRE (essential mitochondrial Ca^2+^ uniporter regulator; Sancak et al., 2013; König et al., 2016) and the complex IV component COX6A1 was not compromised in the absence of ROMO1 (Figs. 4 C and S2, C and D). Yeast Mgr2 has been suggested to function in import of Mgm1. Since OPA1 represents the human orthologue of Mgm1, we analyzed whether ROMO1 was required for OPA1 import and processing. Our analyses showed that import and processing of OPA1 were not affected in the absence of ROMO1 (Fig. S2 E). However, we observed a subtle import defect for the precursor of the early complex IV subunit COX4I-1 (Fig. S2 F). In contrast to COX6A1, COX4I-1 integration into complex IV depends on shuttling of TIM21 between MITRAC and the TIM23 complex (Mick et al., 2012; Fig. 1 A). Taking the observed altered association between TIM21 and TIM23 in the absence of ROMO1 into consideration, we analyzed COX4I-1 integration into early complex IV assembly intermediates (MITRAC complexes) in the absence of ROMO1. Therefore, we imported radiolabeled COX4I-1 into WT and ROMO1-depleted mitochondria and followed complex integration by BN-PAGE. As expected, assembly of COX4I-1 into MITRAC was decreased in the absence of ROMO1 in agreement with reduced TIM21 shuttling (Fig. 4 D). We conclude that ROMO1 is not essential for protein import into mitochondria per se but affects the distribution of TIM21 between the TIM23 and the MITRAC complex, which affects integration of COX4I-1 into the complex IV assembly intermediate.

### ROMO1 is required for import of the *i*-AAA protease YME1L

**Figure 5.**
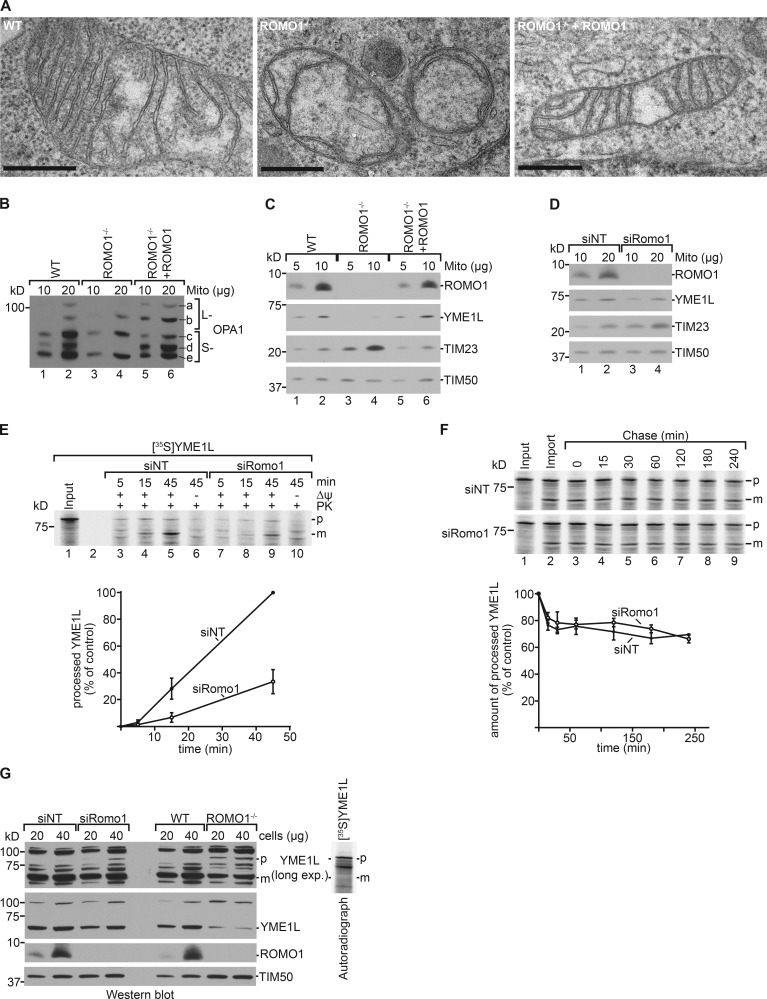
**ROMO1 is required for import of the *i*-AAA protease YME1L. (A)** Mitochondria from WT, ROMO1^−/−^, and ROMO1^−/−^ cells expressing ROMO1 were analyzed by transmission EM. Scale bars: 500 nm. **(B)** Mitochondria from HEK293T WT, ROMO1^−/−^, and ROMO1^−/−^ cells expressing ROMO1 under control of a tetracycline-inducible promoter were lysed and analyzed by SDS-PAGE and Western blot. **(C)** Mitochondria from HEK293T WT, ROMO1^−/−^, and ROMO1^−/−^ cells expressing ROMO1 under control of a tetracycline-inducible promoter were lysed and analyzed by SDS-PAGE and immunoblotting using the indicated antibodies. **(D)** WT cells were treated with siRNA oligonucleotides against *Romo1* or nontargeting control siRNA (NT) for 72 h. Isolated mitochondria were lysed and analyzed by SDS-PAGE and Western blot using the indicated antibodies. **(E)** ^35^S-labeled YME1L was imported into isolated energized mitochondria. Import was stopped at given time points by addition of antimycin A, valinomycin, and oligomycin (AVO). Samples were treated with PK and analyzed by SDS-PAGE and autoradiography. Import of siNT sample at the longest time point was set to 100% (means ± SEM; *n* = 3). p, precursor; m, mature protein. **(F)** ^35^S-labeled YME1L was imported into mitochondria for 45 min (import). After stopping the reaction as before, mitochondria were reisolated, resuspended in import buffer, and incubated further (chase). Samples were taken at indicated time points and analyzed by SDS-PAGE and autoradiography. Amounts of processed YME1L were plotted against time relative to the amount at 0-min chase (set to 100%) (means ± SEM; *n* = 3). p, precursor; m, mature protein. **(G)** WT cells were treated with siRNA oligonucleotides against *Romo1* or nontargeting control siRNA (NT) for 72 h. siNT, siRomo1 as well as WT and ROMO1^−/−^ cells were lysed and analyzed by SDS-PAGE and Western blot. ^35^S-labeled YME1L was synthesized and analyzed by SDS-PAGE and autoradiography.

Previous analyses reported that depletion of ROMO1 led to mitochondrial fragmentation and altered mitochondrial cristae morphology (Norton et al., 2014). Therefore, we assessed mitochondrial membrane morphology of ROMO1^−/−^ cells by EM. Although WT and complemented ROMO1^−/−^ cells displayed similar cristae morphologies, ROMO1^−/−^ cells showed drastically altered cristae morphology (Fig. 5 A). The dynamin-related GTPase OPA1 is crucial for cristae formation of the inner mitochondrial membrane. In its absence, mitochondria display severely disrupted inner membrane morphology (Olichon et al., 2003; Frezza et al., 2006). In mitochondria, OPA1 is processed into five distinct forms by different proteases, OMA1 and YME1L (Griparic et al., 2004; Song et al., 2007; Ehses et al., 2009; Anand et al., 2014; Wai et al., 2015; MacVicar and Langer, 2016). Previous analyses showed that knockdown of ROMO1 resulted in OPA1 processing defects (Norton et al., 2014). In agreement with this, OPA1 processing was significantly altered in ROMO1^−/−^ cells, and OPA1 form d did not accumulate. This defect could be rescued by expressing ROMO1 under a tetracycline-inducible promoter (Fig. 5 B).

Our data show that ROMO1 is a translocase constituent, which affects dynamics of TIM21 and concomitantly complex IV biogenesis. In addition, ROMO1 is required for proper OPA1 processing and thus inner mitochondrial membrane morphology. The absence of OPA1 form d indicated that the proteolytic cleavage of OPA1 by YME1L at site S2 was impaired in the absence of ROMO1 (Fig. 5, A and B). Therefore, we assessed the protein levels of YME1L in ROMO1^−/−^ and siRNA-depleted ROMO1 mitochondria. YME1L could not be detected in ROMO1^−/−^ cells, whereas the YME1L substrate TIM23 accumulated (Fig. 5 C). In contrast, siRNA-mediated depletion of ROMO1 only slightly altered YME1L and TIM23 levels (Fig. 5 D). We thus concluded that prolonged loss of ROMO1 affects the amounts of YME1L, which leads to altered OPA1 processing and concomitantly to inner membrane morphology defects.

How can the absence of a translocase constituent affect the abundance of an inner membrane protease? It seemed conceivable that the reduced levels of YME1L observed in the absence of ROMO1 were due to a specifically decreased import efficiency for YME1L. We tested this hypothesis by in vitro import of YME1L into energized mitochondria from cells treated with siRNA against ROMO1 or control cells. Indeed, the import of YME1L was reduced by 65% in ROMO1-depleted mitochondria (Fig. 5 E). To determine whether the observed lower levels of imported YME1L were directly linked to its import efficiency and not to reduced stability of the imported precursor, we followed the decay of imported YME1L over time in pulse–chase experiments. The stability of imported YME1L did not differ between siRNA control and siRomo1-treated mitochondria (Fig. 5 F). To test which step of import was affected in the absence of ROMO1, Western blot analyses of YME1L were performed on whole cells treated with siNT or siRomo1 and between WT and ROMO1^−/−^ cells. A slower migrating band accumulated in siRomo1 and ROMO1^−/−^ cells, which comigrated with [^35^S]YME1L (Fig. 5 G). Accordingly, loss of ROMO1 leads to accumulation of the YME1L precursor in the cytosol.

Taken together, we found an unexpected link between the mitochondrial protein import machinery and the *i*-AAA protease. ROMO1 is critical for import of YME1L into mitochondria, which fails to accumulate in the absence of ROMO1. The YME1L import defect leads to a reduction of the *i*-AAA protease and concomitantly to altered OPA1 processing and abnormal cristae morphology.

### The presequence of YME1L renders its import dependent on ROMO1

YME1L is anchored to the inner membrane by a single transmembrane span and exposes a large C-terminal domain in the intermembrane space (Hartmann et al., 2016; Shi et al., 2016). This topology differed from the previously tested membrane proteins (see Figs. 4 and S2), and we hypothesized that ROMO1 could be required for efficient import of this long C terminus. To investigate this, we generated C-terminally shortened constructs of YME1L (Fig. S3 A) and performed in vitro import experiments (Fig. S3, B–D). However, import efficiency of these constructs was reduced to the same extent as for full-length YME1L, demonstrating that the C-terminal domain was not responsible for the observed ROMO1 dependence of YME1L import.

Since the transmembrane domain of a protein determines its lateral release from the translocase, we analyzed whether ROMO1 was required for YME1L membrane integration. Therefore, we exchanged the transmembrane domain of YME1L with the corresponding region of COX6A1 (Fig. S3 A), the transport of which was not affected by the absence of ROMO1 (Figs. 4 C and S2 D). However, a transmembrane swap did not alter the ROMO1 dependence of YME1L (Fig. S3 E).

**Figure 6.**
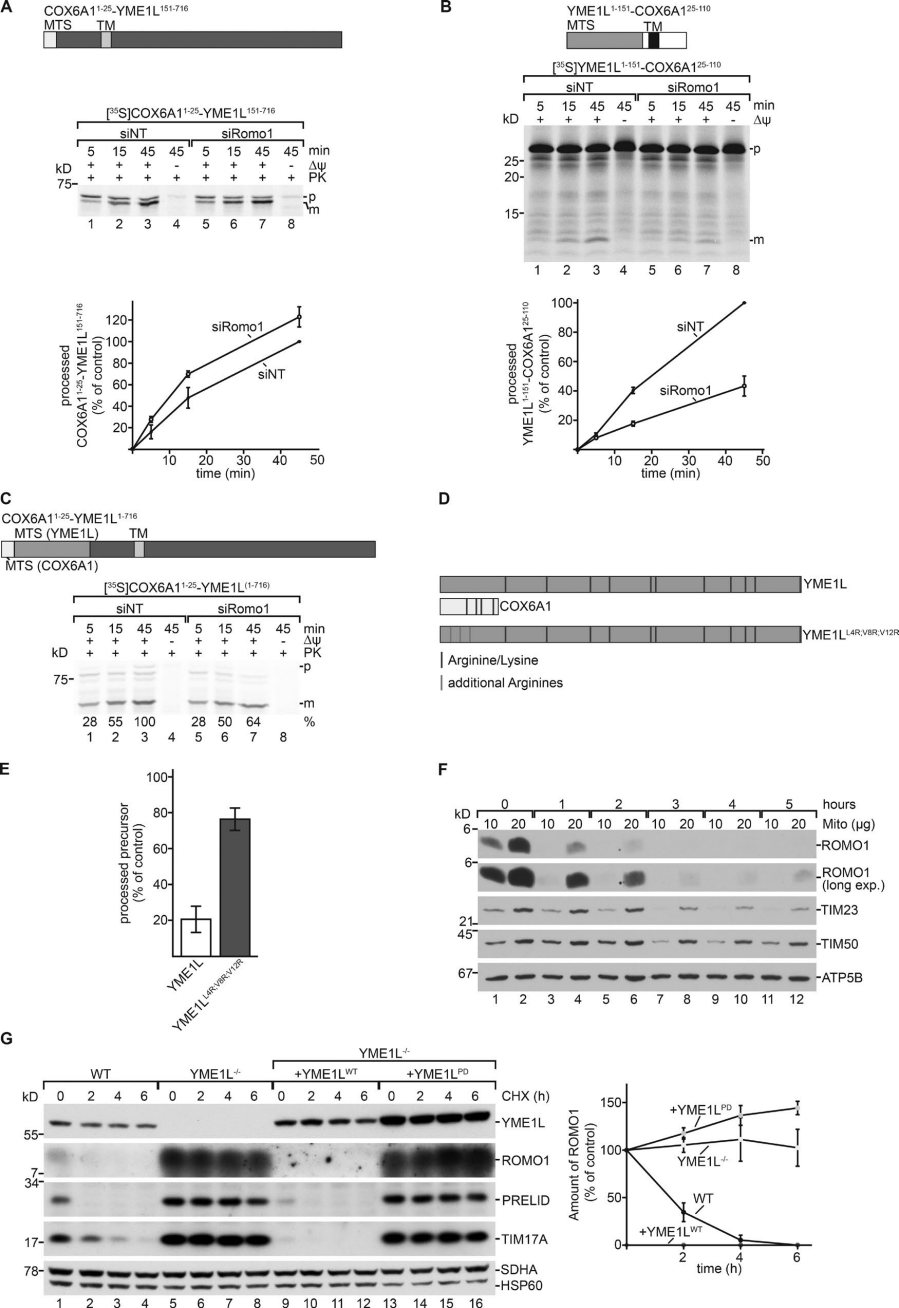
**The presequence of YME1L renders its import dependent on ROMO1. (A and B)** The MTS of COX6A1 (first 25 amino acids) was fused to the mature part of YME1L (151 to end; A). The MTS of YME1L (first 151 amino acids) was fused to the mature part of COX6A1 (25 to end; B). Indicated ^35^S-labeled precursors were imported into isolated energized mitochondria and analyzed by SDS-PAGE and autoradiography. Import of siNT sample at the longest time point was set to 100% (means ± SEM; *n* = 3) is shown. p, precursor; m, mature protein. **(C)** The MTS of COX6A1 (first 25 amino acids) was fused to the precursor form of YME1L (1–716). ^35^S-labeled precursor proteins were imported into isolated energized mitochondria, treated with PK, and analyzed by SDS-PAGE and autoradiography. Import of siNT sample at the longest time point was set to 100%. p, precursor; m, mature protein. **(D)** Schematic representation of arginines and lysines in presequences of YME1L and COX6A1. Schematic depiction of precursor used in E. Three nonpolar amino acids were replaced by arginines as indicated. **(E)** ^35^S-labeled YME1L (Fig. 5 E) and ^35^S-labeled YME1L^L4R;V8R;V12R^ were imported into isolated energized mitochondria and analyzed by SDS-PAGE and autoradiography. Import of respective siNT sample at 15 min was set to 100% (mean ± SEM; *n* = 3). **(F)** HEK293T WT cells were treated with emetine for indicated time points, lysed, and analyzed by SDS-PAGE and immunoblotting. **(G)** HEK293 WT, YME1L^−/−^, and YME1L^−/−^ cells expressing WT or proteolytic-dead YME1L in the presence of tetracycline were treated with cycloheximide (CHX; 100 µg/ml) for the indicated time points. YME1L-dependent degradation of substrates was monitored by SDS-PAGE and immunoblotting. The mean ROMO1 protein band intensity was quantified ± SEM (*n* = 3).

Compared with most mitochondrial proteins, YME1L utilizes a very long presequence of 150 amino acids (Stiburek et al., 2012; Hartmann et al., 2016). To assess whether the long presequence of YME1L led to ROMO1 dependence for import, we swapped the mitochondrial targeting sequences (MTSs) between COX6A1 and YME1L (Fig. 6, A and B). In vitro import of the radiolabeled precursor proteins revealed that import of COX6A1(MTS)-mYME1L became independent of ROMO1, while the import of the YME1L(MTS)-mCOX6A1 fusion construct became dependent on ROMO1 (Fig. 6, A and B). These analyses suggested that an exceptionally long presequence on YME1L might render its import dependent on ROMO1.

To test this idea, we performed a database search to identify further mitochondrial proteins with long presequences (>100 amino acids) and identified LETM1 and COQ8A. After synthesizing radioactively labeled precursors of these proteins, we performed in vitro import assays. Compared with YME1L, only slightly reduced import efficiencies were observed in the absence of ROMO1, indicating that ROMO1 dependence was not a general feature of proteins with long presequences (Fig. S3, F and G). To dissect if the sheer length of the presequence of YME1L leads to ROMO1 dependence for import, the presequence of COX6A1 was fused to the full-length YME1L precursor protein (COX6A1^1–25^-YME1L^1–716^; Fig. 6 C). In vitro import assays revealed that the addition of the COX6A1 presequence improved the import efficiency of YME1L significantly (Fig. 6 C). Therefore, the length of the presequence could not be the determining factor for the import dependence on ROMO1.

Mitochondrial presequences are amphipathic, α-helical segments with a net positive charge due to the presence of arginine and lysine residues. The biophysical and structural properties are critical for recognition by receptors and ΔΨ-driven inner membrane translocation. When comparing the presequences of YME1L and COX6A1, we noticed that the N-terminal portion of the presequence of YME1L lacks positively charged amino acids (Fig. 6 D). This led us to hypothesize that the lack of positive charges at the beginning of the presequence rendered the import of YME1L dependent on ROMO1. Therefore, we replaced three nonpolar amino acids in the N-terminal portion of the presequence by arginines (Fig. 6 D). After synthesizing radioactively labeled precursors, we performed in vitro import assays to assess the import efficiency of YME1L^L4R;V8R;V12R^ in comparison to the authentic YME1L (Fig. 6 E). The addition of positively charged amino acids to the presequence of YME1L alleviated the import defect of YME1L significantly (Fig. 6 E).

In the course of our experiments, we observed that ROMO1 displayed an unusually fast turnover. When we assessed stability of ROMO1 in cells by inhibiting cytosolic translation and followed ROMO1 amounts over time, ROMO1 levels decreased dramatically already after 2 h of inhibited cytosolic translation. Other mitochondrial proteins, such as TIM23, TIM50, and ATP5B, were only marginally affected during this time frame (Fig. 6 F). Accordingly, compared with other subunits of the preprotein translocase, ROMO1 displayed an exceptionally short half-life. Based on this observation and the role of ROMO1 in YME1L import, we assessed whether the *i*-AAA protease YME1L was responsible for ROMO1 turnover. For this, we used a YME1L knockout cell line (YME1L^−/−^). Besides other known YME1L substrates such as TIM17A (Rainbolt et al., 2013) and PRELID (Potting et al., 2013), we observed a drastic accumulation of ROMO1 in the absence of YME1L (Fig. 6 G). Furthermore, ROMO1 was degraded upon reexpression of YME1L^WT^ but accumulated upon expression of a protease-dead version of YME1L.

We conclude that ROMO1 is degraded by YME1L. These experiments thus reveal an intricate feedback regulation of the accumulation of YME1L: while ROMO1 as constituent of the TIM23 complex ensures efficient import of newly synthesized YME1L into mitochondria, allowing its assembly into the *i*-AAA protease, YME1L proteolysis limits the accumulation of ROMO1 and the import of further YME1L precursor proteins.

## Discussion

Although there is a high level of conservation between the mitochondrial translocation machineries of *Saccharomyces cerevisiae* and their human counterparts, recent studies revealed that there are stunning differences in composition and molecular function of translocase constituents (Sokol et al., 2014; Kang et al., 2017, 2018; Vukotic et al., 2017). Here we identified ROMO1 as a new subunit of the human presequence translocase. ROMO1 coisolates together with the core translocase subunits, the channel-forming TIM23 and TIM17 complex, the presequence receptor TIM50, and TIM21. While ROMO1 is dispensable for general import, we find that it is required for the recruitment of TIM21 to the translocation machinery. Based on sequence similarity, ROMO1 was speculated to be related to yeast TIM23 complex subunit Mgr2 (Fig. S1 A; Žárský and Doležal, 2016). However, this idea was not experimentally addressed. Mgr2 has been implicated in quality control during lateral transport of precursor proteins facilitated by the presequence translocase. A loss of Mgr2 accelerates lateral protein transport (Ieva et al., 2014). Here we find that in human mitochondria, a loss of ROMO1 does not affect matrix transport or, in contrast to yeast, inner membrane sorting. However, ROMO1 couples TIM21 to the human translocase and thereby affects its dynamic distribution between MITRAC assembly intermediates and the TIM23 complex. Accordingly, loss of ROMO1 affects the assembly of the early subunit COX4I-1, which is transported to the MITRAC complex by TIM21 (Mick et al., 2012). This finding explains the observed reduced cytochrome *c* oxidase levels upon loss of ROMO1. Similarly, Mgr2 has been implicated in the recruitment of Tim21 to the Tim23 channel in yeast. However, this recruitment appears to be crucial for the assembly of Tim21 rather than respiratory chain biogenesis, as *mgr2*Δ mitochondria do not display OXPHOS defects (Gebert et al., 2012). A recent study suggested that in an artificial membrane system, purified and reconstituted ROMO1 oligomerizes to form ion-conducting channels (Lee et al., 2018). However, it remains to be addressed whether ROMO1 forms ion-conducting channels in vivo. Considering that a fraction of ROMO1 is not translocase associated, it cannot be excluded that, in addition to its role in YME1L import and TIM21 distribution, ROMO1 displays moonlighting function. Based on the yeast studies that Mgr2 can be cross-linked to an arrested precursor (Ieva et al., 2014), it is conceivable that Mgr2 and ROMO1 could be part of the channel-forming portion of the translocase. Accordingly, our findings demonstrate that ROMO1 acts at the TIM23 complex and participates in respiratory chain biogenesis through TIM21. Hence, it can be concluded that the observed increased ROS production in the absence of ROMO1 is rather an indirect effect of loss of ROMO1. In agreement with this idea, siRNA-mediated knockdown of ROMO1 does not promote ROS production but affects TIM21 dynamics.

Unexpectedly, our analyses revealed severely reduced levels of YME1L in ROMO1^−/−^ cells. The protease YME1L participates in processing of the dynamin-like OPA1. Concomitant with the loss of YME1L, we find corresponding OPA1 processing defects and alterations of mitochondrial morphology. Aberrant OPA1 processing and cristae structure have previously been reported under conditions of ROMO1 depletion (Norton et al., 2014). This phenotype is recapitulated in the ROMO1^−/−^ cells but was not apparent under knockdown conditions used here, despite the fact that ROMO1 is effectively depleted. Hence defective processing of OPA1 and altered morphology are caused by the loss of YME1L upon prolonged ROMO1 deficiency and are thus indirectly linked to ROMO1 deficiency. The observed loss of YME1L in the absence of ROMO1 is explained by the observation that ROMO1 is specifically required for import of YME1L into mitochondria. This import defect for YME1L is apparent under ROMO1 knock-down conditions before a loss of YME1L at steady state. The YME1L import defect is linked to its unusually long presequence and can be rescued by replacing the authentic YME1L presequence by the shorter presequence of COX6A1 or by adding the presequence of COX6A1 N-terminally of the YME1L presequence. We find that the low number of positively charged amino acids in the presequence of YME1L are a major reason for its dependence on ROMO1 for import. Adding positively charged amino acids at the beginning of the presequence largely rescued the ROMO1 dependence. However, it remains to be addressed how broad the requirement for ROMO1 is for import of precursors with long and low charged presequences. Ieva et al. (2014) reported altered processing of Mgm1, the yeast homologue of human OPA1, in the absence of Mgr2 and altered mitochondrial morphology. In yeast, Mgm1 is processed by the peptidase Pcp1. However, Pcp1 levels are not affected in *mgr2*Δ mitochondria. Thus, in contrast to human mitochondria, in yeast the observed processing defect of Mgm1 is not due to an altered protease abundance but rather linked to the lipid insertion of the Mgm1 membrane span (Ieva et al., 2014). We show that in contrast to yeast, ROMO1 is not required for import and processing of OPA1.

A surprising observation of our study is that the instability of ROMO1 can be attributed to turnover by the *i*-AAA protease YME1L. A loss of YME1L increases ROMO1 levels drastically in mitochondria. This is the first example for proteolytic control of the composition of a protein translocase to fine-tune its substrate specificity. Hence, the import of YME1L is dependent on ROMO1, and at the same time ROMO1 is turned over by its cargo protein. This reciprocal relationship is unexpected and may indicate that amounts of both YME1L and ROMO1 need to be balanced against each other to maintain physiological steady state levels of both proteins in the cell. This direct link between the translocase component ROMO1 and the protease YME1L, which is involved in OPA1 processing, explains the previously observed heterogeneous phenotypes upon loss of ROMO1 and its link to mitochondrial cristae structure. Thus, ROMO1 provides a coordinated regulation of protein import, OXPHOS assembly (via TIM21), and mitochondrial dynamics (via YME1L).

## Materials and methods

### Cell culture

Human embryonic kidney cell lines (HEK293-Flp-In T-Rex; HEK293T) were cultured in DMEM supplemented with 10% (vol/vol) FBS (Biochrom), 1 mM sodium pyruvate, 2 mM l-glutamine, and 50 µg/ml uridine at 37°C under a 5% CO_2_ humidified atmosphere. Cell growth was measured by Trypan blue exclusion assay. Cells were seeded and stained with 0.04% Trypan blue (Life Technologies) and counted using an automated cell counter. For inhibition of cytosolic translation, medium was supplemented with 20 µg/ml emetine dihydrochloride hydrate (Sigma-Aldrich) or 100 µg/ml cycloheximide before cell lysis with radioimmunoprecipitation assay buffer.

### Generation of knockout and stable cell lines

CRISPR/Cas9 genome editing was used to generate HEK293T cells lacking ROMO1 as previously described (Ran et al., 2013). Briefly, a DNA fragment specific for the human ROMO1 gene was generated and cloned into the pX330 vector using the following primers: forward, 5′-CACCGTTCGTGATGGGTTGCGCCGT-3′; reverse, 5′-AAACACGGCGCAACCCATCACGAAC-3′. Cells were cotransfected with a GFP encoding vector, and GFP-positive cells were single-cell sorted into 96-well dishes. Clones were expanded and screened for the loss of ROMO1 by immunoblotting. Genomic DNA extraction and PCR amplification of the ROMO1 gene, followed by sequencing, confirmed the mutation. HEK293 Flp-In T-Rex cells lacking YME1L and the process used to generate YME1L^−/−^ cells expressing WT or proteolytic dead (E543Q) have been previously described (Saita et al., 2018). HEK293T cells stably expressing C-terminally tagged TIM23^FLAG^, TIM21^FLAG^, TIM50^FLAG^, ROMO1^FLAG^, and ROMO1 without tag under a tetracycline-inducible promoter were generated as described previously (Mick et al., 2012). Briefly, cells were cotransfected with the pcDNA5/FRT/TO vector encoding the appropriate sequence of TIM23^FLAG^, TIM21^FLAG^, TIM50^FLAG^, ROMO1^FLAG^, and ROMO1 as well as pOG44 using GeneJuice (Novagen) as transfection reagent. Hygromycin (200 µg/ml) was applied for selection until single clones could be isolated and screened for protein expression by immunoblotting.

### siRNA constructs and transfection

To generate knockdown cells, HEK293T WT cells were transiently transfected with siRNA oligonucleotides against ROMO1 (5′-UCUGUCCCUUCCCAUCAAU-3′; 8.25 nM; Eurogentec) as well as nontargeting siRNA as control by reverse transfection. Lipofectamine RNAiMAX (Invitrogen) was used as transfection reagent following the protocol provided by the company. Cells were incubated at 37°C under a 5% CO_2_ humidified atmosphere for 72 h.

### Cellular fractionation and isolation of mitochondria

Mitochondria were isolated (adapted from Panov, 2013). Cells were harvested and resuspended in cold isolation buffer (75 mM mannitol, 225 mM sucrose, 10 mM MOPS, pH 7.2, and 1 mM EGTA) with 2 mM PMSF. Harvested cells were incubated in cold hypotonic buffer (100 mM sucrose, 10 mM MOPS, pH 7.2, and 1 mM EGTA) with 2 mM PMSF for 7 min (5 ml/1 g cells). Subsequently, cells were gently homogenized, and cold hypertonic buffer (1.25 M sucrose, 10 mM MOPS, pH 7.2, 1.1 ml/5 ml) was added to homogenized cells before the volume was tripled with isolation buffer containing 2 mM PMSF and 2 mg/ml BSA. After differential centrifugation, pooled mitochondrial pellets were resuspended in isolation buffer.

### In vitro protein synthesis and import and assembly analysis

This was performed as described previously (Lazarou et al., 2009) with modifications. Precursor proteins were synthesized in vitro in the presence of [^35^S]methionine (Hartmann Analytic) using either the Flexi Rabbit Reticulocyte Lysate System (Promega) or the TNT Quick coupled Transcription/Translation Kit (Promega). The manufacturer’s instructions were followed. Radiolabeled protein precursors were incubated with freshly isolated mitochondria in import buffer (250 mM sucrose, 80 mM potassium acetate, 5 mM magnesium acetate, 5 mM methionine, 10 mM sodium succinate, and 20 mM Hepes/KOH, pH 7.4) with added 5 mM ATP. Import reactions were performed at 30–37°C. The membrane potential was dissipated by adding AVO mix (8 µM antimycin, 1 µM valinomycin, and 20 µM oligomycin). Upon import, mitochondria were either treated with 20 µg/ml proteinase K (PK) or directly harvested and washed with SEM-buffer (250 mM sucrose, 1 mM EDTA, and 10 mM MOPS, pH 7.2). To assess the stability of freshly imported precursor proteins, the described import buffer was supplemented with 5 mM creatine phosphate and 0.1 mg/ml creatine kinase, and import reactions were performed as described. Upon SEM wash, mitochondria were further incubated at 37°C and harvested at indicated time points. Samples were analyzed by SDS-PAGE or solubilized for BN-PAGE analysis and analyzed by digital autoradiography using Phosphorimager screens and a Storm 820 scanner (GE Healthcare). Quantifications were performed using ImageQuant TL 7.0 software (GE Healthcare) with a rolling ball background subtraction.

### Affinity purification

Immunoprecipitations were performed as described (Dennerlein et al., 2015) with modifications. Isolated mitochondria or cells (0.5–2 mg) were lysed in solubilization buffer (50 mM Tris/HCl, pH 7.4, 50–150 mM NaCl, 10% glycerol, 1 mM EDTA, and 1% digitonin) and protease inhibitor cocktail (Roche). Lysates were cleared by centrifugation, and the supernatant was applied onto equilibrated anti-FLAG M2 Affinity Gel (Sigma-Aldrich). Beads were washed (50 mM Tris/HCl, pH 7.4, 50–150 mM NaCl, 10% glycerol, 1 mM EDTA, 0.1% digitonin, and protease inhibitor cocktail) and bound proteins eluted with FLAG peptide. Samples were analyzed by SDS-PAGE and immunoblotting.

### Enzymatic assays, membrane potential, and ROS measurements

In-gel activity assays of respiratory chain complexes were performed as described previously (Wittig et al., 2007). Briefly, solubilized complexes were separated on BN-PAGE, and gel strips were incubated in complex I buffer (0.8 mg/ml nitro blue tetrazolium [NBT], and 0.3 mg/ml NADH in 5 mM Tris, pH 7.4), complex II buffer (0.8 mg/ml NBT, 0.2 mM phenazine methosulfate, and 20 mM sodium succinate in 5 mM Tris, pH 7.4), complex IV buffer (1 mg/ml reduced cytochrome *c* and 0.5 mg/ml diaminobenzidine in 50 mM potassium phosphate buffer, pH 7.4), and complex V buffer (14 mM magnesium sulfate, 8 mM ATP, and 0.13% lead nitrate in 35 mM Tris, 220 mM glycine) until precipitations appeared. Activity and relative amount of cytochrome *c* oxidase were analyzed by Complex IV Human Specific Activity Microplate Assay Kit (Mitosciences, Abcam), and the manufacturer’s instructions were followed. A total of 15 µg solubilized cells was loaded per well, and the absorbance at 550 nm was measured. Following incubation with a specific antibody conjugated to alkaline phosphatase, the absorbance at 405 nm was measured. Succinate dehydrogenase activity assay was assessed as described before (Dudek et al., 2016). Briefly, isolated mitochondria were resuspended in hypotonic buffer (5 mM MgCl_2_ in 25 mM potassium phosphate buffer, pH 7.2) to a final concentration of 1 mg/ml. Lysed mitochondria were diluted 1:5 in assay buffer (2.5 µM rotenone, 10 µM antimycin A, 1 mM KCN, and 10 mM sodium succinate in 50 mM potassium phosphate buffer, pH 7.4) and equilibrated to 30°C. To start the reaction, 50 µM coenzyme Q_1_ (CoQ1) was added, and the absorbance at 280 nm was followed to detect the reduction of CoQ1 upon the oxidation of succinate to fumarate. The malate dehydrogenase activity assay was based on Barrie Kitto (1969) and performed as described before (Dudek et al., 2016). Isolated mitochondria were lysed in solubilization buffer (0.5% Triton X-100 in 0.1 M potassium phosphate, pH 7.6) in a final volume of 1 mg/ml, and malate dehydrogenase activity was assayed by diluting solubilized mitochondria 1:40 in assay buffer (0.1 mM NADH and 0.2 mM oxalacetic acid in 100 mM potassium phosphate, pH 7.6) and measuring the disappearance of NADH at 340 nm. Mitochondrial membrane potential was measured using the Membrane Potential Dye JC-1 (Affymetrix eBioscience) according to the manufacturer’s instructions. Cells were incubated with 2 µM JC-1 at 37°C for 15 min, washed with PBS, and resuspended before they were analyzed by fluorescence-activated cell sorting (FACS Canto II; BD Biosciences). An emission shift from red (585 nm) to green (530 nm) was correlated to loss of membrane potential. ROS were measured using MitoSOX Red mitochondrial superoxide indicator (Molecular Probes, Invitrogen), and instructions by the manufacturer were followed. Cells were incubated with 3 µM MitoSOX at 37°C for 10 min, briefly washed with PBS, and resuspended before being processed by fluorescence-activated cell sorting (FACS Canto II, BD Biosciences) at an excitation/emission of 510/580 nm.

### EM

Cells were grown to 95% confluence on Aclar film and fixed for 16 h in 2.5% glutaraldehyde. After a second fixation with 1% osmium tetroxide (3 h), cells were poststained with 0.1% uranyl acetate for 30 min. After dehydration in a graded ethanol series and propylene oxide, the cells were embedded in epoxide resin (Agar 100; Plano). Ultrathin sections were examined using a Philips CM 120 transmission electron microscope, and images were taken with a TemCam F416 CMOS camera (Tietz Video and Image Processing Systems).

### SILAC/MS

SILAC analyses were performed as described (Dennerlein et al., 2015), and immunoaffinity purification of proteins was performed as described above. Samples were pooled in a 1:1 ratio and proteins were separated by PAGE using a 4–12% gradient gel (NuPAGE; Invitrogen). Two biological replicates (two times SILAC forward and reverse labeling) were performed and analyzed for immunoaffinity-purified TIM50 and TIM21, and one biological replicate (forward and reverse) for TIM23. After Coomassie staining, lanes were cut into 23 slices, and proteins were in-gel digested under standard conditions with trypsin. Extracted and dried peptides were dissolved in 2% ACN/0.05% TFA and subjected to liquid chromatography–MS working at standard conditions using an Agilent 1100 LC-system equipped with an in-house packed C_18_ column (ReproSil-Pur 120 C18-AQ, 1.9-µm pore size, 75-µm inner diameter, and 30-cm length; Dr. Maisch GmbH) coupled online to an Orbitrap Velos mass spectrometer (Thermo Fisher Scientific). For data analysis, raw files were processed by MaxQuant (v. 1.3.0.5; Cox and Mann, 2008) with the following settings: multiplicity was set to 2 with Arg10/Lys8 as heavy labels and activated requantify option; trypsin was used as digestion enzyme with maximal two missed cleavage sites; the search was performed against the UniProt-TrEMBL human database (November 2012); oxidation of methionines, carbamidomethylation of cysteines, and acetylation of protein N termini were set as variable modifications, whereas the first two were also included in protein quantification; false discovery rate was set to 0.01. Data interpretation was performed by using the software platform Perseus (v. 1.6.0.7; Cox and Mann, 2012). For generating the scatter plots, the following ratio cutoffs were applied: a value of 1 (log2) for each forward and reverse experiment and additionally a value of 1.5 (log2) for the average of both conditions. Annotated data after MaxQuant analysis are listed in Table S1. Columns labeled 21/23/50 H/L describe identified proteins after SILAC and FLAG immunoisolations of TIM21^FLAG^/TIM23^FLAG^/TIM50^FLAG^ Heavy/Light, respectively.

### Immunoblotting

Proteins separated by SDS-PAGE were transferred onto PVDF membranes (Millipore) by semidry blotting. Primary antibodies used were ROMO1 (ProteinTech, 24200-1-AP, rabbit), ROMO1 (Origene, TA505580, mouse), OPA1 (BD Biosciences, 612607, mouse), YME1L (ProteinTech, 11510-1-AP, rabbit), SDHA (Cell Signaling, 5839, rabbit), SDHA (Invitrogen, 459200, mouse), TIM17A (ProteinTech, 11189-1-AP, rabbit), TIM17A (GTX108280), TIM17B (ProteinTech, 11062-1-AP, rabbit), TOM20 (ProteinTech, 11802-1-AP, rabbit), mtHSP60 (StressMarq, SMC-110, mouse), PRELID1 (Abnova, H00027166-M01, mouse), and home-made ROMO1, FLAG, TIM50, TIM21, TIM23, MITRAC12, Rieske, ATP5B, COX1, TOM70, MITRAC7, COX6A1, COX6C, NDUFA4, COX4I-1, and LETM1 (all rabbit). Membranes were probed with either an infrared secondary antibody or secondary antibody coupled to HRP. Signals were scanned or visualized using the enhanced chemiluminescence detection kit (GE Healthcare) on x-ray films. Quantifications were performed using ImageQuant TL 7.0 software (GE Healthcare) with a rolling ball background subtraction.

### BN-PAGE analysis

BN-PAGE was performed as described previously (Mick et al., 2012). Mitochondria were solubilized in 1% digitonin-containing buffer (20 mM Tris-HCl, pH 7.4, 0.1 mM EDTA, 50 mM NaCl, 10% glycerol [wt/vol], and 1 mM PMSF) for 20 min on ice at a concentration of 1 mg/ml. Insoluble material was removed by centrifugation, and the supernatant was added to sample loading buffer (0.5% Coomassie Brilliant Blue G-250, 50 mM 6-aminocaproic acid, and 10 mM Bis-Tris/HCl, pH 7.0). Electrophoresis was performed on 4–13%, 4–14%, and 2.5–10% gradient gels as described (Wittig et al., 2006), followed by immunoblotting or autoradiography. Primary antibodies used were SDHA (Cell Signaling, 5839, rabbit) and home-made Rieske, COX1, VDAC, NDUFA9, COX4I-1, ATP5B, MITRAC12, and NDUFA10 (all rabbit).

### Statistical analysis

Data in the bar and line graphs are represented as means ± SEM. Statistically significant differences between groups were calculated with the help of Prism 5 software (GraphPad Software) by one-tailed unpaired Student’s *t* test.

### Online supplemental material

Fig. S1 shows a sequence alignment of Mgr2 and ROMO1 as well as the sequence information of the ROMO1^−/−^ cell line. Fig. S2 shows additional precursor imports extending Fig. 4, C and D. Fig. S3 relates to Fig. 6 and shows schemes of YME1L constructs and their imports as well as further precursors with a long presequence. Table S1 relates to Fig. 1, C–E, and shows the annotated data of MaxQuant analysis after SILAC analysis.

## Supplementary Material

Supplemental Material (PDF)

Table S1 (Excel)
