# Supplementary material for: ROMO1 is a constituent of the human presequence translocase required for YME1L protease import
Source: J Cell Biol. 2019 Feb 4;218(2):598–614. doi: 10.1083/jcb.201806093 (PMC6363466; doi:10.1083/jcb.201806093)
Supplement: Supplemental Material (PDF) [file JCB_201806093_sm.pdf]

Supplemental material

Richter et al., https://doi.org/10.1083/jcb.201806093

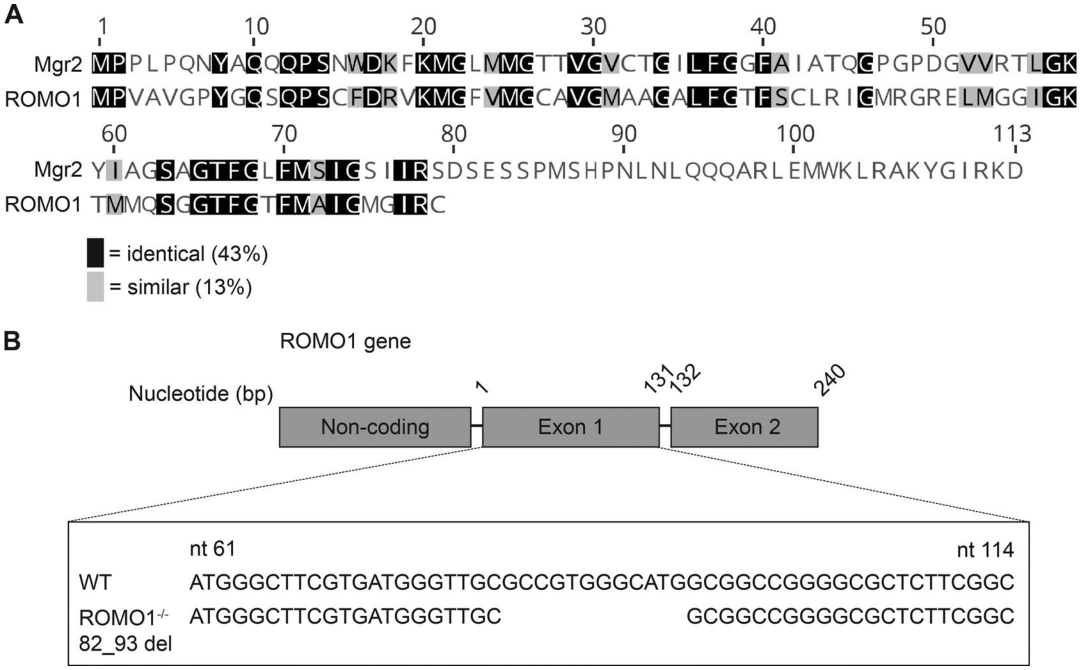

Figure S1. **Generation of a ROMO1$^{-/-}$ cell line. (A)** Sequence alignment of Mgr2 and ROMO1. **(B)** Genomic DNA of WT and ROMO1$^{-/-}$ cells was isolated, and ROMO1 ORF was amplified by PCR and sequenced.

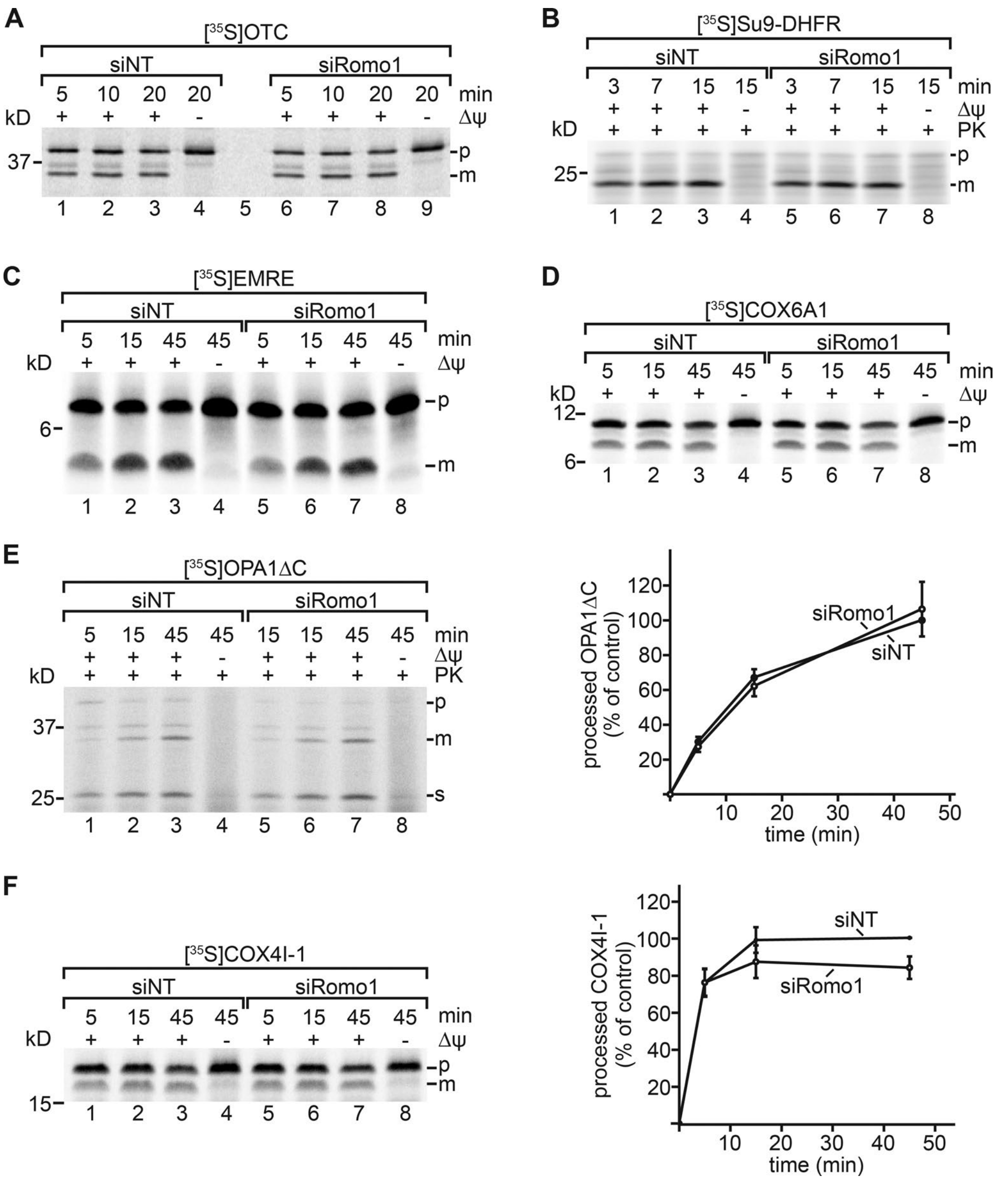

Figure S2. **ROMO1 is not essential for protein import into mitochondria per se. (A–D)** Indicated ^{35}S-labeled precursors were imported into isolated energized mitochondria. Import was stopped at given time points by addition of antimycin A, valinomycin, and oligomycin (AVO). Samples were treated with PK where stated and analyzed by SDS-PAGE and autoradiography. p, precursor; m, mature protein. **(E)** ^{35}S-labeled OPA1ΔC (OPA1 splice variant 7, 1–394) was imported into isolated energized mitochondria as above. Samples were treated with PK and analyzed by SDS-PAGE and autoradiography. Import of siNT sample at the longest time point was set to 100% (means ± SEM; *n* = 3). p, precursor; m, mature protein; s, processed OPA1 at S1. **(F)** ^{35}S-labeled COX4I-1 was imported into isolated energized mitochondria. Import was stopped at given time points by addition of AVO. Samples were analyzed by SDS-PAGE and autoradiography. Import of siNT sample at the longest time point was set to 100% (mean ± SEM; *n* = 3). p, precursor; m, mature protein.

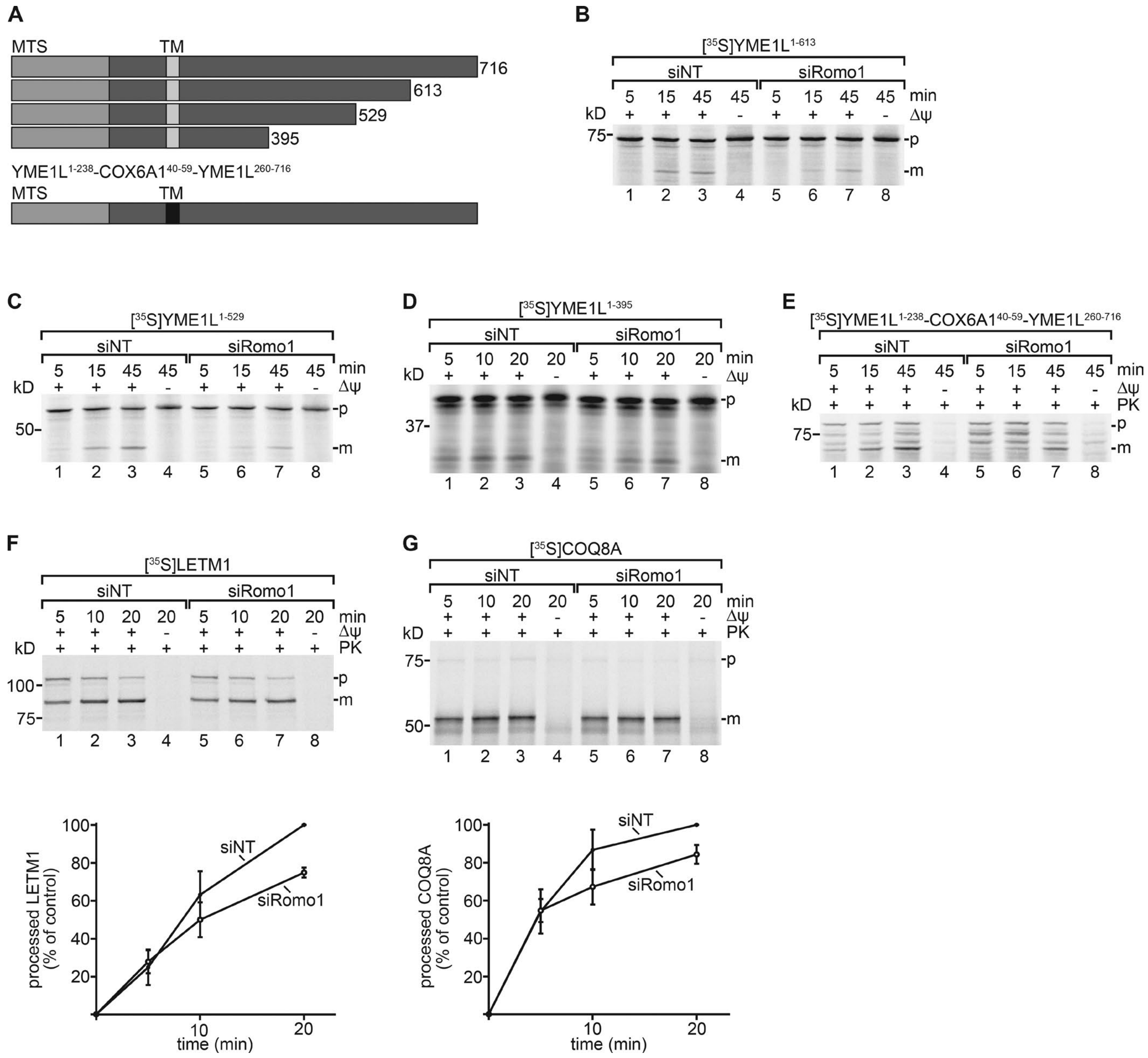

Figure S3. **Molecular dissection of import dependence on ROMO1. (A)** Schematic depiction of precursors used in B–E. The full-length protein (716) was shortened C-terminally as shown. The transmembrane domain (TM) of YME1L was exchanged for the transmembrane domain of COX6A1. TM, transmembrane domain. **(B–G)** Indicated ^{35}S-labeled precursors were imported into isolated energized mitochondria. Samples were treated with PK where stated and analyzed by SDS-PAGE and autoradiography. Import of siNT sample at longest time point was set to 100% (means ± SEM; *n* = 3) is shown. p, precursor; m, mature protein.

Table S1 is provided as an Excel file and shows annotated data of MaxQuant analysis after SILAC analysis following immunoaffinity purification of TIM23^{FLAG}, TIM21^{FLAG}, and TIM50^{FLAG}.
